# Sexual Functioning of Patients with Inflammatory Bowel Disease

**DOI:** 10.3390/jcm15062379

**Published:** 2026-03-20

**Authors:** Marta Kotkowicz-Szczur, Lidia Kisielewska, Rafal Kisielewski, Maciej Kierzkiewicz, Jaroslaw Kierkus, Edyta Szymanska

**Affiliations:** 1 Department of Gastroenterology, Hepatology, Feeding Disorders and Paediatrics, The Children’s Memorial Health Institute, 04-730 Warsaw, Poland; m.kotkowicz@ipczd.pl (M.K.-S.); j.kierkus@ipczd.pl (J.K.); 2 Department of Gynecologic Oncology, The Centre of Oncology, 15-027 Bialystok, Poland; l.kisielewska@gmail.com (L.K.); r.kisielewskia@onkologia.bialystok (R.K.); 3 Department of Internal Medicine and Gastroenterology, Międzyleski Specialist Hospital, 04-749 Warsaw, Poland; m.kierzkiewiczo@o2.pl; 4 Department of Internal Medicine, Faculty of Medicine, Collegium Medicum, Cardinal Stefan Wyszyński University, 01-938 Warsaw, Poland

**Keywords:** inflammatory bowel disease, sexuality, relationship, chronic disease, sexual disorders

## Abstract

**Background/Objectives**: Chronic diseases, such as inflammatory bowel disease (IBD), influence patients’ sexuality. Therefore, the aim of this study was to analyze the sexual functioning (SF) of patients with IBD. **Methods:** We perform a prospective survey study on male and female individuals with IBD using an anonymous questionnaire including 60 inquiries concerning patients’ intimate relationships and SF. The following statistical tests were used: chi-square test of independence, Spearman’s rank correlation coefficient, and Wilcoxon and Mann–Whitney U tests. A significance level of *p* = 0.05 was assumed as statistically significant. **Results**: There were 110 respondents with IBD (41% with Crohn’s disease and 57% with ulcerative colitis): 52 women (47%) and 58 men (53%), with a mean age of between 31 and 40 (45%). In 34% of respondents, the assessment of satisfaction with sex after diagnosis decreased, and the difference was statistically significant (*p* = 0.007). Statistically significant correlations were found between IBD clinical activity and the impact of the disease on sexual desire (*p* < 0.001), the need for sex after diagnosis (*p* < 0.001), satisfaction with sex after diagnosis (*p* = 0.003), the average frequency of intercourse (*p* = 0.004), the average duration of intercourse after diagnosis (*p* = 0.001), feeling guilty in the sexual sphere due to the disease (*p* = 0.006), assessment of one’s attractiveness since diagnosis (*p* = 0.032), and change in the partner’s erotic perception after diagnosis (*p* < 0.001). The more aggressive the course of the disease, the more negative the impact on patients’ sexuality. **Conclusions:** The diagnosis of IBD has a negative impact on patients’ SF—disease flare leads to a decrease in sexual needs, worse experiences and negative body image.

## 1. Introduction

Inflammatory bowel diseases (IBDs), including Crohn’s disease (CD) and ulcerative colitis (UC), are chronic, progressive and disabling conditions. They can occur from early childhood to late adulthood, although the peak incidence of CD is between 20 and 30 years of age and UC between 30 and 40 years of age [1]. These disorders are characterized by a recurrent course, unpredictable exacerbations, hospitalizations, the need for surgery and a deterioration in the patients’ quality of life (QoL) [2].

In this context, IBD patients are at increased risk of psychological burden. Conditions such as anxiety and/or depression are more common in these individuals than in healthy ones [3]. The research has also shown that patients with IBD may have impaired body image, raising concerns about their mental stability and social and sexual life [4,5].

Previous studies have demonstrated that sexual dysfunction (SD), defined as a persistent or recurring sexual problem causing marked personal distress or interpersonal difficulties, is more common in patients with IBD than in the general population [6,7]. According to available data, between one- and two-thirds of patients report SD associated with their underlying disease [8,9]. Thus, disorders of sexual function (SF) should not be ignored in individuals with IBD, and they should be screened for both psychological and sexual problems, with necessary treatments provided as soon as possible [10,11].

The aim of this study was to assess the influence of intestinal disease and its activity on the SF of Polish individuals with IBD.

Although common questionnaires are used in empirical studies around the world, they do not effectively address the unique symptoms and issues associated with SD in IBD patients. Therefore, the authors developed an original questionnaire taking into account aspects of the disease in the context of sexual activities.

## 2. Patients and Methods

An anonymous self-prepared survey for male and female patients with IBD containing 60 inquiries concerning sexual life was performed. Participation in the survey was voluntary.

The first part of the questionnaire included general and demographic information: respondent’s age, area of residence, education, economic status, and type and status of relationship.

The second part was related to sexual life: frequency of sexual intercourse, quality of sexual intercourse, satisfaction, SD, and self-perception of one’s attractiveness and appearance or partner’s perception.

The third part contained information on the impact of IBD on patients’ SF.

The final part concerned the patient’s general attitude toward sexuality and any sexual counseling provided by their physician.

The study was approved by the local Bioethical Committee—Approval No 51/KBE/2025.

Written informed consent was obtained from parents and all individual participants included in the study.

### Statistics

The significance of differences in the distribution of variables measured on an ordinal scale between two groups was checked using the Mann–Whitney U test.

Correlations between variables were checked using Spearman’s rank correlation coefficient.

The significance of differences in assessments before and after diagnosis was checked using the Wilcoxon test.

The significance of the relationship between nominal variables was checked using the chi-square test of independence.

In statistical analyses, the significance level was *p* = 0.05. Analyses were performed using the SPSS program Statististica StatSoft pl 13.1.

## 3. Results

### 3.1. Patients

The survey involved 110 patients with IBD—52 women (47%) and 58 men (53%). Most of them were aged between 31 and 40 (45%) and between 41 and 50 (21%).

The majority of respondents lived in district (43%) and voivodeship (32%) cities.

The respondents most often assessed their economic status as good (45%).

Nearly 88% of respondents were in a relationship; 43% of them were married, 35% were in a civil partnership, and 5% in another kind of relationship. The relationship length was most often 5 to 10 years (35%).

Nearly 41% of respondents were diagnosed with CD, 57% of them with UC, and 2% with IBD unclassified (IBDU). Most often, the disease was diagnosed between the ages of 13 and 18 (39%) or between the ages of 19 and 25 (35%).

The course of the disease so far was most often moderate (57%) and less often mild (36%) or severe (13%). The majority of respondents (46%) were in incomplete remission, 36% of them were in full remission and 16% had an exacerbation of the disease.

Respondents were treated with 5-ASA (amino salicylate) agents (28%), anti-TNF alfa drug (17%) or another biological treatment (38%).

Less than 6% of respondents underwent surgery due to IBD, and 2% of them had a stoma. Extraintestinal symptoms of the underlying disease were present in 8% of patients.

Respondents most often assessed their well-being related to the disease as moderate (57%) and less often as bad (17%) or good (26%) (Table 1).

### 3.2. Sexual Life of Respondents with IBD

Most men did not observe the impact of the disease on puberty (79%), 10% of men did, and 9% did not have night or morning erections.

The female respondents most often had their first menstrual period at the age of 14 (25%) or 13 (27%), slightly less often at the age of 15 (17%). The majority of women had a regular period. Of the women, 29% did not have regular periods, and 6% had menstrual disorders.

Most often, the respondents had had between one and three sexual partners (53%). Nearly 56% of them used contraceptive methods. The most frequently used contraception was a condom (22%), and contraceptive coils (10%), pills (10%) and patches (1%) were used less often.

**Figure 1:**
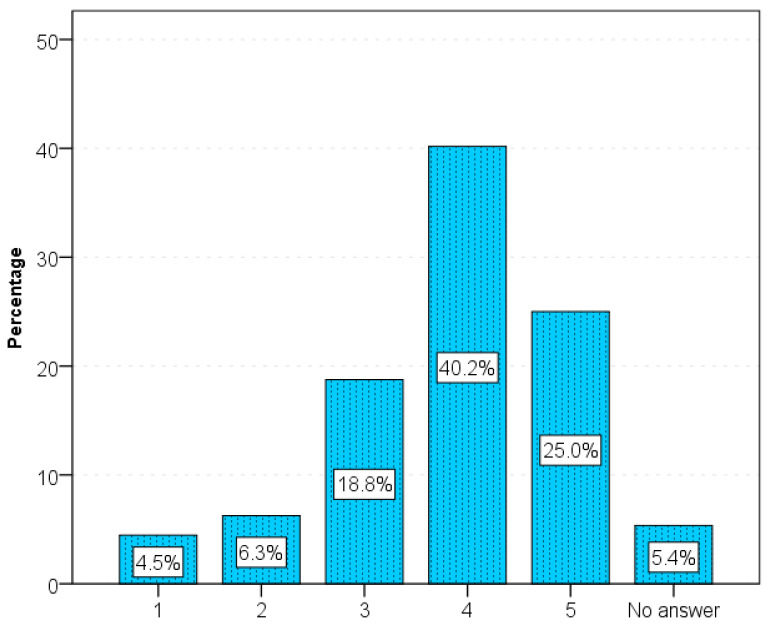
Sexually satisfaction before and after diagnosis of IBD.

Underlying intestinal disease affected the desire for sexual activity rarely (28%), moderately often (34%) or not at all (31%). After the diagnosis of IBD, most people had a moderate need for intercourse and sex (66%) (Figure 1).

**Figure 2:**
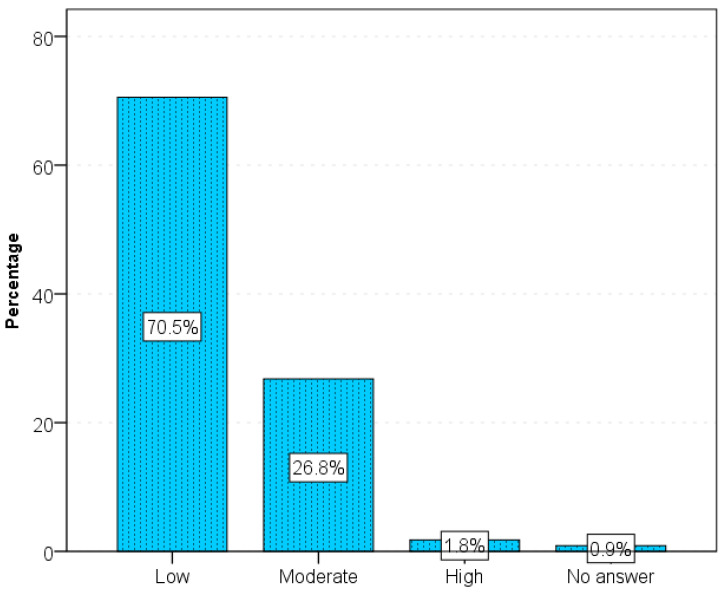
Sexual needs before and after diagnosis

The respondents had to assess their satisfaction with sex using a five-point scale both before and after the diagnosis of IBD. Before the diagnosis, respondents rated satisfaction as 5 (42%), and less often as 4 (21%), while after the diagnosis it was 4 (35%), less often as 5 (25%), and rarely as 3 (14%), 2 (10%) and 1 (11%) (Figure 2).

Before diagnosis, respondents most often had sex twice a month (28%) or three times a week (24%). After diagnosis, intercourse was mostly had twice a month (34%), and a large percentage had sex less than twice a month (14%), three times a week (19%) or two to three times a week (15%).

Thirty-eight percent of respondents had less desire for sex than before their diagnosis, and 54% of them did not observe any change on that matter.

In nearly 43% of patients, the exacerbation of IBD had no impact on their sexuality, while 14% felt a lesser need for sex.

Most respondents did not have to give up sexual activity due to illness (46%). The remaining respondents gave up during exacerbations (36%) or repeatedly, regardless of disease activity (15%).

The majority of respondents believed that the disease had affected their sexual relations in the last year (76%). Symptoms of IBD most often had a moderate impact (39%) on sexual activity. The most common intestinal or extraintestinal symptoms of IBD that most adversely affected sexual activity were abdominal pain (11%) and diarrhea (7%).

Most people had not noticed a change in their intimate or emotional relationship with their partner since the diagnosis of IBD (53%) and a large percentage of people had noticed such a change (34%). Also, in the majority of cases, the diagnosis did not change the respondent’s attitude towards sex (62%); however, 32% of respondents had noticed such a change.

The majority of respondents rated their attractiveness worse than before the diagnosis (48%); only 6% of them rated it the same way.

More than half of the patients claimed that the diagnosis of IBD did not have a negative impact on achieving orgasm (51%).

Before the diagnosis of IBD, SD was experienced by nearly 16% of the surveyed people, and after being diagnosed with IBD this rate increased to 18%.

Nearly 28% of respondents believed that the medications they took had a negative impact on their sexuality or fertility.

Twenty eight percent of patients were afraid of the deterioration of sexual and intimate relationships with their partner when IBD exacerbations occurred.

### 3.3. Attitude Toward Sexuality

Only 34% of respondents claimed that their gastroenterologist discussed the topic of sexuality and fertility in IBD and answered them freely when asked about these issues. Over 29% of patients felt embarrassed and had difficulty talking freely about sexuality with their gastroenterologist; however, over 46% of them expected the physician to talk about this issue.

## 4. Discussion

There is ongoing research into the impact of IBD on intimacy, sexuality, and sexual relationships. As previously mentioned, many factors can negatively impact SF in chronic intestinal diseases, such as symptoms, perianal conditions, body image, surgery, and drug side effects [12].

Nardone et al. reported in their study that the prevalence of SD in IBD patients was 50.6% (95% CI = 40.8–60.5%; I2 = 96.3%), with an OR = 2.94 (95% CI = 1.99–4.35, I2 = 73.4) compared to healthy controls [13]. Furthermore, it was higher in patients with active disease than in those with inactive disease (75.1% vs. 34.2%; OR = 9.65, 95% CI = 1.02–91.33, I2 = 95.5%). These outcomes are consistent with our results, since we also observed that IBD diagnosis, especially disease activity, had a statistically significant negative impact on patients’ sexuality.

In the review by Ma et al., depression, disease activity and surgery were the most commonly cited disease-related factors affecting sexual health in men [14]. 

However, Mules et al. demonstrated that objective measures of disease activity were not associated with SD or erectile dysfunction (ED) in patients with IBD [15]. In their study, symptoms of severe depression (odds ratio, 5.77; 95% confidence interval, 1.59–20.94) were connected with SD in women, and severe anxiety (odds ratio, 15.62; 95% confidence interval, 1.74–140.23) was associated with ED in men [10].

In a multicenter, national-level study by Roseira et al., patients with IBD had impaired patient-perceived sexual quality of life (SQoL) compared with healthy controls. Age, widow status, and depression were independent predictors of SQoL in men with IBD, whereas in women, depression was the only independent predictor [16]. Emotional and self-esteem issues were the main concerns reported by IBD patients regarding sexual health.

Similarly, the study by Riviere et al. showed that 54% of women experienced SD and 43% of men experienced ED. These rates were significantly higher than in healthy controls, with results mostly driven by psychological factors and independent of disease severity [6].

In the review by Mantzouranis et al., impaired SF was reported in general cohorts of IBD patients; furthermore, depression was a consistent negative predictive factor across studies [17].

Again, mood disorders appear to be the main factor influencing SF, and more frequent occurrence of depression in chronic diseases has been proven [18].

Zhang et al. demonstrated that SD occurred frequently in the IBD population [19]. Operations, depression, disease activity, comorbidities, age  <  50 years, and the need for corticosteroids were risk factors for SD in IBD patients.

A meta-analysis by Zhao et al. revealed that IBD was significantly associated with an elevated risk of SD in both male (seven studies, RR = 1.41, 95% CI, 1.09–1.81, *p* = 0.008; heterogeneity: I2 = 80.2%, *p* < 0.001) and female subjects (five studies, RR = 1.76, 95% CI, 1.28–2.42, *p* < 0.001; heterogeneity: I2 = 69.6%, *p* = 0.011) [20].

In our study, almost one fifth of patients experienced SD after their IBD diagnosis, which was higher than before the diagnosis, and the majority of them (76%) believed that the disease had affected their sexual relations.

In a study conducted by Marin et al., half of the female and one-third of the male patients considered that both sexual desire and satisfaction worsened after IBD diagnosis [21]. Compared to controls, both men and women with IBD showed significantly lower scores in sexual function indexes. These outcomes are consistent with our results—patients had lower sexual desire, felt less need for sex after IBD diagnosis, and had less frequent sexual intercourse per month.

Similarly, a systemic review and meta-analysis by Chen et al. revealed that IBD was associated with worse SF [22]. It had significant impact on erectile function and satisfaction for male individuals and had impact on most sub-domains of SF for female individuals.

A study by Danish authors resulted in similar observations, showing that patients with active disease, stoma, or perianal involvement face more sexual challenges than those without IBD [23].

In a review by Leenhardt et al., pregnancy was a major concern for patients with IBD [24].

According to our survey, nearly 28% of respondents believed that the medications they took had a negative impact on their sexuality or fertility.

An alarming observation is the fact that almost one-third of patients felt embarrassed and had difficulty talking freely about sexuality with their gastroenterologist; however, over 46% of them expected their physician to talk about this issue. This outcome should remind both gastroenterologists and general practitioners to be aware of the effect of IBD on SF and address this issue.

The advantage of this study is the use of a self-developed original questionnaire taking into account aspects of the disease in the context of sexual life. As far as we know, it is one of the few studies on sexuality in the Polish population of IBD patients.

The disadvantage of our survey is the limited number of participants— it is still not sufficient; therefore, further research is needed.

### Suggestions on How to Improve SD

Since the key factor influencing patients’ sexuality is their clinical state, it is very important to provide medical management to maintain remission, upgrade medication as soon as needed and use the best tailored therapy. Another important issue is psychological support—chronic diseases affect all aspects of the patient’s life, and the lives of their family, thus impacting on their QoL, including the quality of their sexual life. Therefore, a psychologist should be the part of medical team taking care of IBD patients and be available to support them.

Finally, when SD occurs, there are medications that can be used for either erectile dysfunction, preterm ejaculation or hormonal disorders, as well as specialist consultations, such as those with a sexologist, gynecologist or endocrinologist [25].

## 5. Conclusions

After diagnosis of IBD, patients’ sexual life worsens and disease activity strongly affects their SF. The exacerbation of IBD causes a decrease in sexual needs, makes sexual experiences worse and negatively affects body image. Physicians should be aware of the potential negative impact of active IBD on patients’ sexual heath, discuss this issue with them, and provide appropriate help.

## Figures and Tables

**Table 1:** Socio-demographic data.

		Number	%
Age (y.o)	31–40	10	8.9%
	41–50	25	22.3%
	51–60	60	53.6%
	>60	17	15.2%
Education	Basic	2	1.8%
	Vocational, technical	22	19.6%
	General secondary	30	26.8%
	Higher first cycle (bachelor’s degree)	10	8.9%
	Higher second cycle (master’s degree)	46	41.1%
	No answer	2	1.8%
Domicile	Village	30	26.8%
	Municipal town	16	14.3%
	County town	36	32.1%
	Regional town	29	25.9%
	No answer	1	0.9%
Economic status	Low	3	2.7%
	Satisfactory	23	20.5%
	Good	69	61.6%
	Very good	14	12.5%
	No answer	3	2.7%
Relationship status	Civil partnership	12	10.7%
	Marriage	89	79.5%
	Other	11	9.8%
Length of the relationship (years)	<2	2	1.8%
	2–5	8	7.1%
	5–10	6	5.4%
	>10	93	83.0%
	No answer	3	2.7%
Length of intercourse with current partner/husband (years)	<2 L	4	3.6%
	2–5	8	7.1%
	5–10	7	6.3%
	>10	90	80.4%
	No answer	3	2.7%
Number of sexual partners so far	0	3	2.7%
	1–3	94	83.9%
	4–8	10	8.9%
	9–13	1	0.9%
	No answer	4	3.6%

Abbreviation. Years of age—y.o.

## Data Availability

The data that support the findings of this study are openly available. The authors confirm that the data supporting the findings of this study are available in this article.
